# Genomic Data Reveal *Toxoplasma gondii* Differentiation Mutants Are Also Impaired with Respect to Switching into a Novel Extracellular Tachyzoite State

**DOI:** 10.1371/journal.pone.0014463

**Published:** 2010-12-30

**Authors:** Pamela J. Lescault, Ann B. Thompson, Veerupaxagouda Patil, Dario Lirussi, Amanda Burton, Juan Margarit, Jeffrey Bond, Mariana Matrajt

**Affiliations:** Department of Microbiology and Molecular Genetics, University of Vermont, Burlington, Vermont, United States of America; University of Missouri-Kansas City, United States of America

## Abstract

*Toxoplasma gondii* pathogenesis includes the invasion of host cells by extracellular parasites, replication of intracellular tachyzoites, and differentiation to a latent bradyzoite stage. We present the analysis of seven novel *T. gondii* insertional mutants that do not undergo normal differentiation to bradyzoites. Microarray quantification of the variation in genome-wide RNA levels for each parasite line and times after induction allowed us to describe states in the normal differentiation process, to analyze mutant lines in the context of these states, and to identify genes that may have roles in initiating the transition from tachyzoite to bradyzoite. Gene expression patterns in wild-type parasites undergoing differentiation suggest a novel extracellular state within the tachyzoite stage. All mutant lines exhibit aberrant regulation of bradyzoite gene expression and notably some of the mutant lines appear to exhibit high proportions of the intracellular tachyzoite state regardless of whether they are intracellular or extracellular. In addition to the genes identified by the insertional mutagenesis screen, mixture model analysis allowed us to identify a small number of genes, in mutants, for which expression patterns could not be accounted for using the three parasite states – genes that may play a mechanistic role in switching from the tachyzoite to bradyzoite stage.

## Introduction

*Toxoplasma gondii* is an obligate intracellular pathogen capable of infecting any nucleated mammalian cell. It reproduces both sexually and asexually where the sexual cycle only occurs in cats while the asexual cycle can occur in a wide variety of intermediate hosts, including humans [1]. In most cases infection is rapidly controlled by the host's cellular immune response, leaving a latent infection. However, reactivation of latent bradyzoites in immunocompromised hosts can lead to fatal encephalitis [2]. Primary infection by tachyzoites can also cause severe abnormalities in the developing fetus [3]. The asexual cycle comprises two developmental stages: rapidly growing tachyzoites and latent, encysted bradyzoites. During the course of infection, tachyzoites disseminate throughout the body where a fraction differentiate to form encysted bradyzoites in muscle and brain, in response to host immune defense [4]. During the transition from tachyzoites to bradyzoites, growth rate is greatly reduced and differentiation-specific markers are induced leading to the establishment of encysted bradyzoites [5]. Tachyzoites and bradyzoites express distinct subsets of surface antigen related sequences (SRSs). Stage specific expression of SRSs is important for parasite persistence and host immune evasion [6], for instance, four tandemly arranged genes encoding bradyzoite SRSs, SAG2CDXY, have been implicated in maintaining a chronic infection in the brain [7].

Due to *T. gondii*'*s* ability to develop into latent bradyzoites in response to immune system attack, it is a challenge to successfully treat this parasitic infection, as there are no current drugs against the encysted bradyzoite form [1]. Given that the asexual cycle is central for the pathogenicity of *T. gondii*, it is important to gain a better understanding of the molecular events that govern this process in order to identify novel drug targets.

Although it is known that the host immune response is responsible for triggering differentiation, very little is known about the molecular environment that induces bradyzoite differentiation *in vivo*. *In vitro* a variety of stress conditions induce bradyzoite formation; including heat shock, alkaline shock, oxidative stress, and pyrimidine starvation [8]. The phosphorylation of *T. gondii* initiation factor-2α has been linked to stress responses and development of bradyzoites [9]. Changes in host cell transcription can directly induce bradyzoite specific gene expression [10], showing that *T. gondii* can also sense signals inside the host cell.

The analysis of expressed sequence tag (EST) assemblies from tachyzoite and bradyzoite cDNA libraries, microarray analysis, and serial analysis of gene expression (SAGE) have identified several stage-specific genes [11]–[13]. Analysis of the *T. gondii* genome reveals a small number of conventional transcription factors, suggesting an important role for the chromatin-remodeling machinery. In *T. gondii*, methylation and acetylation of histones are landmarks of active promoters [14]. In addition, histone-modifying complexes have also been linked to differentiation [15]. More recently, Behnke *et al*., identified and mapped *cis*-acting elements in several bradyzoite promoters that confer basal and regulated expression [16], similar to other eukaryotes. Their data show that conventional promoter mechanisms work with the chromatin-remodeling machinery to regulate bradyzoite gene expression, suggesting that transcription initiation is an important regulatory mechanism during the tachyzoite to bradyzoite transition [16].

Bradyzoite differentiation mutants have been generated in several laboratories [17]–[19]. Here we present the analysis of seven insertional mutants that do not undergo normal bradyzoite differentiation. Whole genome expression profiling was carried out using the newly developed Affymetrix ToxoGeneChip (GeneChip Tgondiia520372) in order to analyze the ∼8,000 predicted genes in the *T. gondii* genome of mutants and wild-type, allowing for full-scale expression profiling during bradyzoite differentiation *in vitro*.

We report the generation, phenotypic and transcriptomic analysis of seven bradyzoite differentiation mutants. We propose there is an additional state, extracellular tachyzoites, which is equally distinct from both intracellular tachyzoites and bradyzoites. Two mutants are able to switch between extracellular tachyzoites and intracellular tachyzoites but are unable to form bradyzoites. The other 5 mutants are delayed in switching between extracellular tachyzoites and intracellular tachyzoites, behaving like intracellular tachyzoites regardless of whether they are extracellular or whether they have received the differentiation stimulus.

## Results

### Genome-wide expression patterns suggest a distinct extracellular tachyzoite state

In order to expand our knowledge of the gene expression changes that occur during normal bradyzoite differentiaion, RNA from wild-type parasites was extracted and hybridized to the ToxoGeneChip. We harvested extracellular tachyzoites from freshly lysed fibroblasts (ET, 0 h), intracellular tachyzoites (IT, 24 h post-invasion) and parasites subjected to bradyzoite growth conditions for 72 h (B72, 72 h of induction). The data is archived at NCBI GEO under Series GSE23174. Quantitative real-time PCR validation experiments confirm the microarray results (Figures S1 and S2).

**Figure 1:**
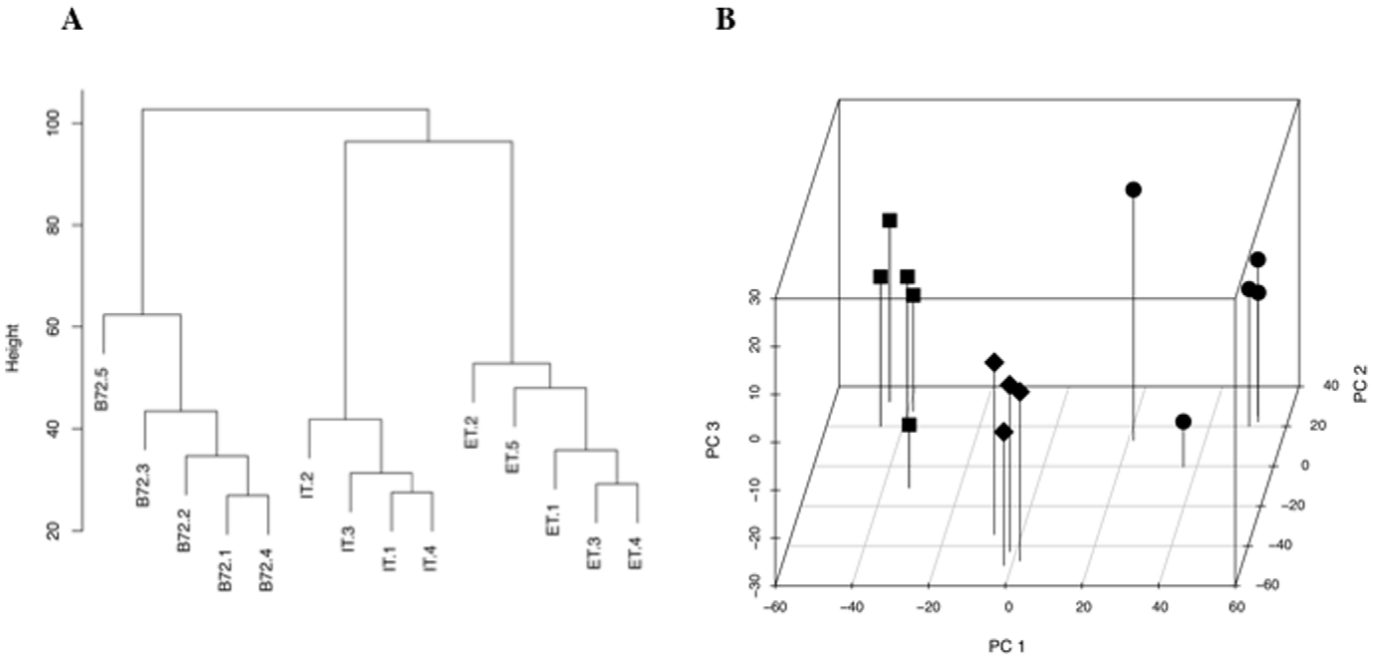
Genome-wide expression suggests three distinct states in the *T. gondii* wild-type asexual cycle. A) Hierarchical clustering carried out with wild-type replicate samples. Replicate samples of extracellular tachyzoites: ET.1–5; intracellular tachyzoites: IT.1–4; and bradyzoites: B72.1–5. B) Principal Component Analysis (PCA) plot of wild-type ET, IT and B72 replicate samples. Principal Component Analysis captures a high proportion of the variation across samples with a small number of parameters, allowing representation of sample proximity in three dimensions. This sample-based method permits to visualize sample-sample distances (i.e., which samples behave most like each other, and most different from each other). ET- circles, IT, diamonds, and B72- squares.

Genome-wide expression statistics (8,058 probe sets representing 7,764 genes) were obtained for extracellular tachyzoites (ET), intracellular tachyzoites (IT), and bradyzoites 72 hours post induction (B72). We find that the difference in expression between ET and IT (RMSD  = 0.83, fold change  = 1.8; p<0.008, see materials and methods) is comparable to the difference (0.89, 1.9; p<0.008) between IT and B72. This is consistent with hierarchical clustering (Figure 1A), which shows variation within ET, IT, and B72 sample groups is smaller than variation between sample groups as well as exhibiting similar distances between sample groups. It is also consistent with principal component analysis (Figure 1B). The first two principal components, which capture 82% of the variance (Figure S3), appear to be dominated by differences between sample groups while principal component 3 captures variation largely within sample groups.

To examine *changes* in expression of each gene in the genome (from 0 h to 72 h post induction), we calculated the difference in expression level of each pair of expression statistics (IT – ET, B72 – ET, and B72 – IT). For example, the genes that are up-regulated more than 2 fold after 72 h of bradyzoite induction (compared with IT) have a logarithm base 2 fold change greater than or equal to one (log_2_FC ≥1 or B72 – IT ≥ 1) or, equivalently, a fold change greater than or equal to two (i.e., bradyzoite over intracellular tachyzoite expression, B72^I^/IT^I^ ≥2; the equivalent B72/IT ratio is of *intensity* at 72 h over intensity at 24 h for a given gene, whereas, when we refer to B72 - IT, we use *expression statistics*).

**Figure 2:**
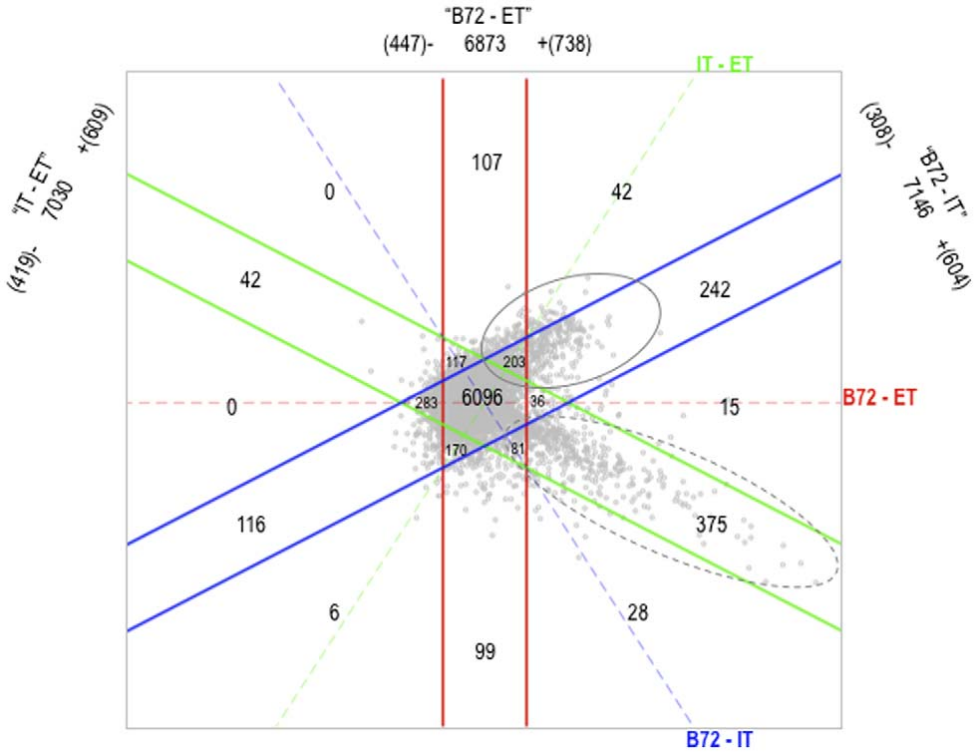
Three-way differential expression (DE) plot shows three distinct states in the *T. gondii* wild-type asexual cycle. This plot slices the 8058 predicted *T. gondii* genes, (each point represents a gene) into specific gene sets that reflect all possible comparisons (IT – ET, B72 – ET, and B72 – IT). Each comparison is associated with a dashed axis, which captures the magnitude of differential expression associated with the comparison, and two solid lines, which bound the set of genes differentially expressed less than 2-fold by the comparison. Of the 8,058 probe sets, 6,096 (central hexagon) are not changed more than 2-fold between any of the 3 states. The extracellular tachyzoite-specific genes lie at the intersection between genes that are up-regulated in extracellular tachyzoites compared to intracellular tachyzoites (IT-ET ≤−1; n = 419, below the green solid line) and genes that are upregulated in extracellular tachyzoites compared to bradyzoites (B72-ET ≤−1; n = 447, to the left of the red solid line), which total 122 genes. The intracellular tachyzoite-specific genes lie at the intersection between genes that are up-regulated in intracellular tachyzoites compared to extracellular tachyzoites (IT-ET ≥1; n = 609, above the green solid line) and genes that are up-regulated in intracellular tachyzoites compared to bradyzoites (B72-IT ≤−1; n = 308, above the blue solid line), which total 149 genes. And, the bradyzoite-specific genes lie at the intersection between genes that are up-regulated in bradyzoites compared to extracellular tachyzoites (B72-ET≥1; n = 738, to the right of the red solid line) and genes that are up-regulated in bradyzoites compared to intracellular tachyzoites (B72-IT≥1; n = 604, below the blue solid line), which total 418 genes.

Differential expression of genes with respect to the three comparisons (IT – ET, B72 – ET, and B72 – IT) can be visualized simultaneously using a ternary plot (Figure 2). The gene ontology (GO) terms for genes that are up-regulated at least 2-fold in each state (ET, IT or B72) compared to the 2 other states (e.g., ET ≥2-fold compared to both IT and B72) are listed in Dataset S1. Notably, this plot shows there are two major groups of differentially expressed genes (indicated in Figure 2 with ovals). The first major group of genes lies in the solid oval; these genes are induced after host cell invasion (up-regulated in IT compared to ET). The second major group of genes lies in the dashed oval; these genes are induced during bradyzoite formation (up-regulated in B72 compared to both ET and IT).

Many of the genes that are up-regulated in IT (solid oval) compared to ET encode proteins involved in cell growth processes, such as, 3′,5′ cyclic nucleotide phosphodiesterase activity (GO:0004114; p = 4.1e-06) and DNA replication (GO:0006260; p = 0.002), consistent with the biology of intracellular tachyzoites which grow and replicate rapidly.

The genes up-regulated in B72 (dashed oval) compared to ET and IT are enriched for the biosynthetic process GO term (GO:009058; p<0.005). This GO term is defined as the arm of metabolism that involves transformation of simpler products into more complex products. This is consistent with the biology of bradyzoites that have to store energy for long-term survival and for use during the more energy-requiring stages (i.e., the rapidly dividing intracellular tachyzoites). For example, bradyzoites are characterized by the presence of a large number of amylopectin granules (storage polysaccharide), and these granules are thought to serve as energy storage and energy that is required during the transition of developmental stages [20].

Rhoptries are unique secretory organelles shared by all apicomplexan parasites, and the proteins secreted from these organelles (ROP proteins) play critical roles in parasite invasion, growth and virulence [21]. There are 31/44 rhoptry genes that are differentially expressed at least 2-fold between ET, IT, and B72 (Table 1). For example, ROP 16 expression is highest in IT, while ROP 18 expression is highest in IT and B72. ROPs 16 and 18 are kinases that are secreted into the host cell upon invasion and constitute important virulence factors [22]. Recently, it was shown that the ROP family of proteins is under positive selection, is coccidian-specific, and it also shows an extraordinary degree of differential expression between strains [23]. For instance, ROP38 which is 64 times up-regulated in VEG strain compared to RH strain, was shown to alter the expression of ∼1200 host genes. An RH strain engineered to express high levels of ROP38 (comparable to the levels of VEG strain) suppressed most of the transcriptional changes induced by RH strain [23]. Interestingly, ROP38 is also one of few ROP proteins that is induced during bradyzoite differentiation (Table 1).

**Table 1:** Rhoptry genes are differentially expressed across the three wild-type states.*

	Up-regulated in ET (below green solid lines)				
ToxoDB r5.3	r4.3	Description	ET	IT	B72
TGME49_063220	55.m05046	Rhoptry kinase family protein ROP21	9.61	8.06	8.51
TGME49_039600	49.m03159	Rhoptry kinase family protein ROP23	9.31	7.96	11.12
TGME49_027010	42.m03546	Rhoptry kinase family protein ROP30	10.46	8.68	9.44
TGME49_104740	540.m00203	Rhoptry kinase family protein ROP35	12.46	11.42	11.63
TGME49_081790	74.m00442	Rhoptry kinase family protein ROP45	8.19	6.99	7.47
TGME49_115940	583.m05718	rhoptry protein, putative	9.46	8.46	9.16

	Up-regulated in IT (solid oval)				
ToxoDB r5.3	r4.3	Description	ET	IT	B72
TGME49_027810	42.m03584	Rhoptry kinase family protein ROP11	10.19	13.07	12.48
TGME49_062730	55.m08219	Rhoptry kinase family protein ROP16	9.45	11.10	10.69
TGME49_058580	55.m08191	Rhoptry kinase family protein ROP17	11.23	12.96	12.60
TGME49_005250	20.m03896	Rhoptry kinase family protein ROP18	10.63	12.91	12.87
TGME49_058230	55.m04748	Rhoptry kinase family protein ROP20	6.85	8.11	8.82
TGME49_052360	52.m01543	Rhoptry kinase family protein ROP24	9.89	11.87	11.09
TGME49_042230	49.m03276	Rhoptry kinase family protein ROP29	6.89	10.23	9.83
TGME49_015780	33.m01398	Rhoptry kinase family protein ROP2A	10.48	13.03	12.27
TGME49_094560	83.m01271	Rhoptry kinase family protein ROP37	8.38	10.28	9.82
TGME49_062050	55.m08224	Rhoptry kinase family protein ROP39	11.12	12.32	12.23
TGME49_095110	83.m02145	Rhoptry kinase family protein ROP4/7	9.93	12.42	12.11
TGME49_091960	80.m02343	Rhoptry kinase family protein ROP40	9.95	12.59	11.99
TGME49_108080	551.m00238	Rhoptry kinase family protein ROP5	11.13	13.03	12.85
TGME49_015770	33.m00005	Rhoptry kinase family protein ROP8	8.49	10.12	10.56
TGME49_100100	145.m00331	rhoptry neck protein 2	8.15	10.97	10.12
TGME49_058660	55.m00092	rhoptry protein 6	10.35	12.99	12.64
TGME49_109590	583.m00003	rhoptry protein, putative	10.32	12.60	12.11
TGME49_115220	583.m00692	rhoptry protein, putative	8.24	10.78	10.70
TGME49_115210	583.m00694	rhoptry protein, putative	7.36	9.80	9.32

	Up-regulated in B72 (dashed oval)				
ToxoDB r5.3	r4.3	Description	ET	IT	B72
TGME49_039600	49.m03159	Rhoptry kinase family protein ROP23	9.31	7.96	11.12
TGME49_058370	55.m10291	Rhoptry kinase family protein ROP28	4.92	5.18	8.89
TGME49_042110	49.m03275	Rhoptry kinase family protein ROP38	7.44	7.66	9.51
TGME49_009980	25.m01903	Rhoptry kinase family protein ROP42	10.66	9.95	12.53
TGME49_030470	44.m02599	Rhoptry kinase family protein ROP46	8.20	7.99	9.29
TGME49_053330	52.m01578	Rhoptry kinase family protein, truncated	4.97	5.97	10.75

*Expression statistics for ET, IT, and B72.

Taken together, the results shown in figures 1 and 2, suggest a novel extracellular state within the tachyzoite stage. We propose the *T. gondii* asexual cycle includes 3 states: extracellular tachyzoites, intracellular tachyzoites and bradyzoites.

### Generation and phenotypic analysis of novel T. gondii differentiation mutants

We took advantage of parasites that lack an endogenous copy of the hypoxanthine/xanthine/guanine phosphoribosyltransferase (HXGPRT) gene (useful as both a positive or negative selectable marker) and reintroduced this marker under the control of an early bradyzoite-specific promoter [17]. Using this stable parasite line we expected that, following mutagenesis, parasites in which a positive regulator of the bradyzoite differentiation signaling cascade is inactivated should be resistant to HXGPRT negative selection under bradyzoite growth conditions.

Insertional mutagenesis was carried out using the parental parasite line described above with a vector that integrates at random, followed by bradyzoite induction *in vitro* and negative selection to isolate differentiation mutants. Approximately 20% of stably transformed parasites resulted in mutants defective in bradyzoite differentiation. Here we describe the isolation of seven mutants that are unable to undergo normal bradyzoite differentiation. Southern blot analysis shows that each mutant has been disrupted in a single, distinct locus (Figure S4). Additionally, we identified the genomic DNA flanking the insertion site in five out of seven mutants and gene expression level for each disrupted gene is shown (Table 2). The expression profile of the genes surrounding the insertion point in each of the mutants is shown in Table S1.

**Table 2:** The disrupted loci in the bradyzoite differentiation mutants*.

Mutant	Annotation ToxoDB r5.3	Notes	ToxoDB r4.3	Fold change(B72 vs ET)	
				WT	Mutant
B7	TGME49_038110 (replication factor A protein 3 domain-containing)	insertion 345 bp upstream from TGME49_038110	None		
P11	TGME49_013640 (splicing factor, arginie/serine-rich, putative)	insertion inside exon1	31.m00914	0.8	1.1
7K	TGME49_004420 (oocyst wall protein COWP, putative)	insertion inside exon2	20.m03865	2.7	1.3
11K	TGME49_025010 (hypothetical)	insertion 237 bp downstream from TGME49_025010	42.m03398	1.7	1.2
12K	Identification pending				
13P	Identification pending				
11P	TGME49_049190 (hypothetical) **	insertion inside exon2	50.m03282	1.1	1.3

*Plasmid rescue and inverse PCR (see Materials and Methods) was carried out to identify the *T. gondii* genomic DNA flanking the insertion sites. Flanking regions were cloned, sequenced and aligned using BLAST in ToxoDB (http://toxoDB.org/). Annotation of the physically disrupted gene (or gene closest to the insertion site) is shown. The annotation for ToxoDB r4.3 is also shown as the ToxoGeneChip was built using the 4.3 release. Southern-blots confirm each mutant has been disrupted in a different locus (Figure S4).

**This gene was annotated as AP2 domain transcription factor XII-6.

**Figure 3:**
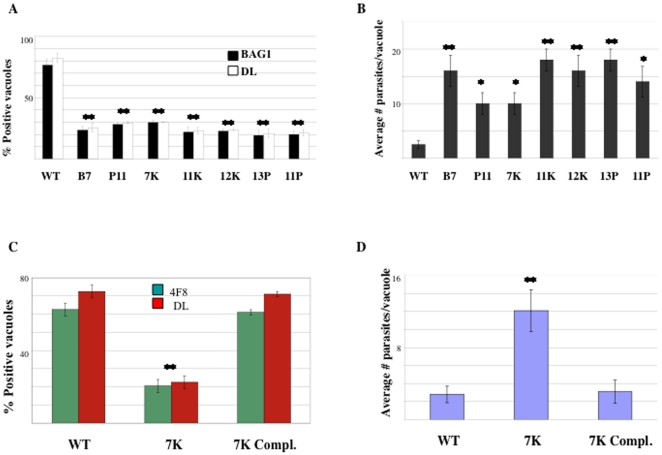
Mutants show defects in bradyzoite-specific gene expression and growth rate. Confluent human foreskin fibroblasts were infected with wild-type and mutant parasites and subjected to bradyzoite induction conditions for 72 hours. A) Immunofluorescence Assays (IFAs) were carried out for bradyzoite antigen 1 (BAG1) and *Dolichos lection* (DL). The presence/absence of marker expression was counted for each parasite line, in triplicate experiments. B) Proliferation after 72 h of induction was measured by counting the number of parasites/vacuole in triplicate experiments. C) Immunofluorescence Assays were carried out for bradyzoite antigens 4F8 and DL. The presence/absence of marker expression was counted for each parasite line, in triplicate experiments. D) Proliferation after 72 h of induction was measured by counting the number of parasites/vacuole in triplicate experiments. The significance of the data was determined by Student's t-test (* p<0.05 and **p<0.01).

Bradyzoites are readily distinguished from tachyzoites based on distinctive biological characteristics. The hallmarks of bradyzoite differentiation are the expression of bradyzoite specific markers and reduced replication rate [8]. In order to confirm that these mutants have defects in cyst formation, we measured bradyzoite marker expression and growth under bradyzoite induction conditions. We quantified the expression level of the major bradyzoite antigen, BAG1, a low molecular weight heat shock protein [24] and the presence of *Dolichos biflorus* lectin (DL), a marker that binds to the cyst wall [25]. All seven mutants have a strong defect in the expression of these bradyzoite markers. After 72 hours of bradyzoite induction *in vitro*, ∼80% of wild-type parasite vacuoles express BAG1 and DL while only 20–30% of mutant parasites express these bradyzoite markers (Figure 3A). These results also show that each mutant population has a ‘leaky’ phenotype, with 20%–30% of each mutant population able to form bradyzoites under differentiation conditions. Considering the significance of bradyzoite formation for the pathogenesis of *T. gondii*, it is very likely that the control of bradyzoite differentiation is a multigenic phenomenon with more than one control point, and therefore disruption of a single gene is unable to completely abolish this process.

Each *T. gondii* parasite establishes a distinct intracellular vacuole inside the host cell within which its progeny replicates with a cell cycle of ∼7 hours, under tachyzoite growth conditions [26]. In contrast, upon differentiation stimulus, the parasites slow down their replication rate continuously, until they stop replicating in mature cysts. *In vitro*, tachyzoites completely lyse the host cell monolayer after 72 hours of growth, while bradyzoites never lyse the host monolayer. We observed little to no growth rate differences between wild-type and mutant parasites or between mutants under tachyzoite growth conditions (data not shown). After 72 hours of bradyzoite induction, wild-type parasites slow down their replication rate, with the average number of parasites per vacuole being ∼2 (Figure 3B). In contrast, mutants contain ∼16 parasites per vacuole (B7, 11K, 12K, 13P and 11P) or ∼10 parasites per vacuole (P11 and 7K). Under bradyzoite conditions, all mutant populations continue growing faster than wild-type until they completely lyse the host cell monolayer, while wild-type never lyses out.

Four mutants (B7, P11, 11K, and 7K) were successfully complemented showing that the physically disrupted genes are responsible for each mutant's phenotype. Here we describe, in detail, the complementation experiments for mutant 7K, as one example of the direct link between mutation and phenotype. The disrupted gene in mutant 7K was identified by inverse PCR and confirmed by southern blot analysis. The mutagenesis vector integrated into exon 2 of 20.m03865 (TGME49_004420), a gene predicted to encode an oocyst wall protein. To confirm that this locus is responsible for the phenotype observed in mutant 7K, mutant parasites were transfected with a cosmid containing a large genomic fragment including the 20.m03865 (TGME49_004420) wild-type locus. A cosmid was used in order to capture all possible genomic regulatory regions for 20.m03865 (TGME49_004420). After transfection, stable parasite lines were obtained, cloned, and assayed for their ability to form bradyzoites. Complementing mutant 7K with the wild-type locus restores the ability of the mutant to differentiate: the complemented mutant expresses bradyzoite markers to the same extent as wild-type parasites (Figure 3C) and slows down replication rate to wild-type levels under bradyzoite induction conditions (Figure 3D). The cosmid used for complementation contains 4 genes in addition to 20.m03865 (TGME49_004420) and this gene is the only gene contained within the complementation cosmid that has altered expression levels in mutant 7K; wild-type parasites up-regulate this gene 3 fold after bradyzoite induction while it's expression is unaffected by induction conditions in mutant 7K (Table 3). These results show that 20.m03865 (TGME49_004420) is responsible for the phenotype of mutant 7K.

**Table 3:** Expression profile of genes present in the cosmid used for complementation.

Gene ID		Fold Change(B72 vs ET)WT	Fold Change(B72 vs ET)7K
ToxoDB r4.3	ToxoDB r5.3		
20.m05911	TGME49_004440	0.6	0.8
20.m05909	TGME49_004430	0.7	0.8
**20.m03865**	**TGME49_004420**	**2.7**	**1.3**
20.m03864	TGME49_004410	1.0	1.0
20.m00382	TGME49_004400	0.9	1.1

### Bradyzoite differentiation mutants are also impaired in the switch to the bradyzoite cell cycle

In order to examine possible perturbations of the cell cycle caused by the genes disrupted in the mutant parasites, we carried out immunofluorescence assays (IFAs) with the cell cycle markers IMC1 and Centrin [27]. Wild-type and mutant tachyzoites were synchronized using pyrrolidine dithiocarbamate (PDTC) as described by Conde de Felipe et al. [28], and IFAs carried out every two hours for an entire cell cycle (∼7 hours). In addition, we induced the parasites to differentiate and performed IFAs at 12 h, 24 h and 48 h post bradyzoite induction.

Bradyzoite differentiation is characterized by slow and asynchronous replication, and a combination of endodyogeny and endopolygeny [29]. In contrast, tachyzoites normally divide synchronously by endodyogeny and, as a result, the parasitophorous vacuoles contain 2, 4, 8, 16, 32, 64 parasites, while bradyzoite vacuoles often contain odd number of parasites.

Under tachyzoite growth conditions, wild-type and mutant parasites divide synchronously with similar replication rate, and no differences were observed between wild-type and mutants (data not shown, but very similar to Figure S5).

**Figure 4:**
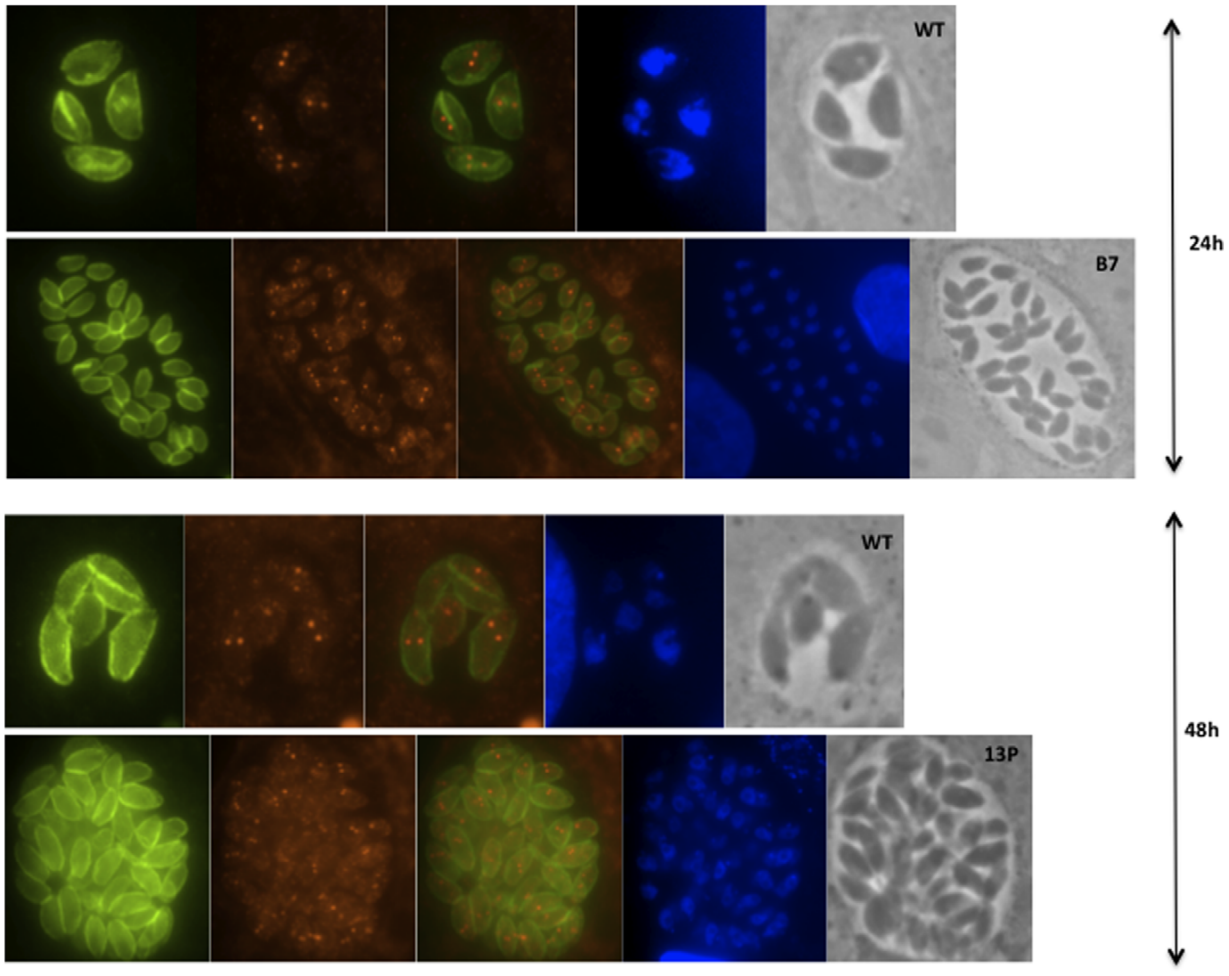
Mutant parasites have a tachyzoite-like cell cycle under differentiation conditions. IFAs carried out at 24 h and 48 h post bradyzoite induction. Parasites were stained with antibodies against the cell cycle markers IMC1 (green), Centrin (red) and dapi (blue).

After 12 h of bradyzoite induction both wild-type and mutant parasites divide synchronously (Figure S5), but after 24 h of bradyzoite growth, wild-type parasites start to slow down their replication rate and ∼30% of the vacuoles contain odd number of parasites, for example, see Figure 4 where only one of the four parasites in the vacuole is dividing into 3 parasites. After 48 h of growth under bradyzoite conditions, wild-type parasites continue to slow down their replication and the percentage of vacuoles with odd number of parasites increases. In contrast, mutant parasites divide mainly synchronously and faster at all time points after bradyzoite induction (Figure 4). These results suggest that mutant parasites continue under the tachyzoite cell cycle in bradyzoite growth conditions and the genes disrupted in the mutants are likely to block bradyzoite differentiation upstream of the decision to change the cell cycle, i.e. the signal that leads to the change from the tachyzoite to bradyzoite cycle maybe blocked in these mutants.

### Differential expression scatter plots of all genes for wild-type versus each mutant reveal differences between groups of genes and parasite lines

**Figure 5:**
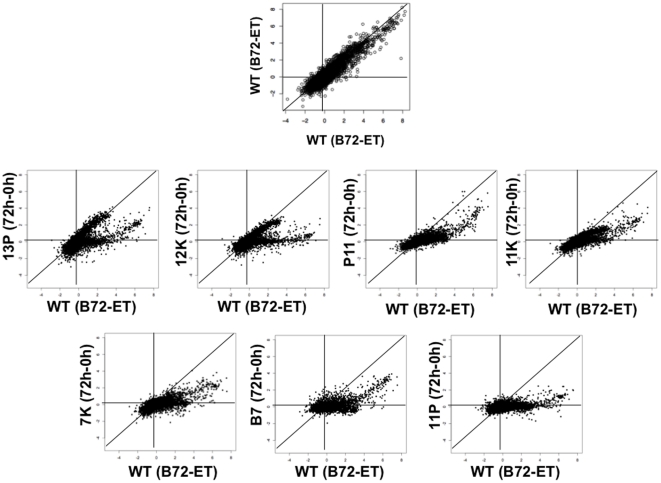
Differential expression (DE) scatter plots of wild-type versus each mutant suggest different classes of behavior among genes and parasite lines. The differential expression for wild-type (B72 - ET) was plotted against the differential expression for each mutant (72 h–0 h). (ET): wild-type extracellular tachyzoites, (B72): wild-type parasites 72 h post-bradyzoite differentiation conditions, (0 h): mutant extracellular tachyzoites, (72 h): mutant parasites 72 h post-bradyzoite differentiation conditions. Data represents the average of three replicate experiments per replicate sample/per condition. The top plot shows wild-type replicate 1 versus wild-type replicate 2 as an example of what the shape of the plot would look like if two samples were nearly identical (biological replicates).

In order to gain a better understanding of the impact of the insertional mutations on global gene expression in response to the differentiation stimulus (*i.e.*, the treatment response by genotype interaction), we generated differential expression scatter plots. Extracellular parasites from freshly lysed fibroblasts were harvested for the seven mutant parasite lines (0 h) and mutant parasites subjected to bradyzoite differentiation conditions for 72 h were harvested (72 h). Differential expression for each mutant (72 h–0 h) was plotted against wild-type (B72 – ET) (Figure 5). The differential expression (DE) scatter plots allow to visualize which gene(s) in the differentiation mutants behave like wild-type and which gene(s) differ in bradyzoite induction levels compared to wild-type. Three distinct groups of genes are observed: (1) genes that respond comparably to induction conditions in the wild-type and mutants (genes that cluster along the diagonal line); (2) genes that fail to respond to induction conditions in the mutants (genes that cluster along the horizontal line); and (3) genes that respond to a lesser extent to bradyzoite induction conditions in the mutant parasites (genes that fall between the diagonal and horizontal lines). The expression levels of many genes in this latter group reflect the leakiness of the mutant lines, since a proportion of each mutant parasite population (20–30%, Figure. 3A) are capable of differentiating into bradyzoites.

Scatter plots suggest different classes of behavior among mutants. Mutants, 13P and 12K, show a split, sideways “Y”-shaped plot, where some genes respond comparably to wild-type while others fail to respond to bradyzoite induction conditions. In contrast, mutants P11, 11K, 7K, B7, and 11P show little to no response to bradyzoite induction conditions (most of the genes cluster along the horizontal line).

### Cluster analysis of genes up-regulated in bradyzoites reveals sets of co-expressed genes whose expression is significantly affected in the mutant parasite lines

Because each mutant line is defective in inducing wild-type-like gene expression in response to bradyzoite induction conditions, we clustered genes that are up-regulated in bradyzoites (compared to ET and to IT) in order to identify differences between mutants with respect to these genes. We used a distance measure based on the Pearson correlation coefficient and according to this distance measure, two genes that fall on the same line through the origin in all DE scatter plots (Figure 5) will superimpose when their expression statistics are centered and scaled, i.e., if gene A and gene B both become up-regulated in response to bradyzoite induction conditions, but to different magnitudes, they will superimpose on each other after centering and scaling. This method allowed us to group genes based on similar expression pattern across all wild-type and mutant parasite lines.

**Figure 6:**
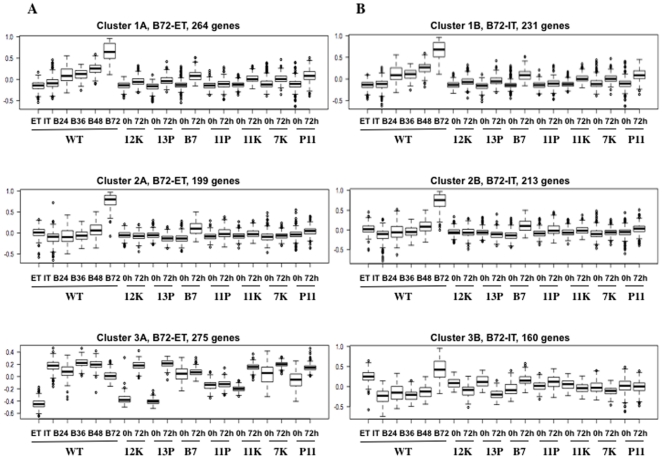
Cluster analysis of genes that are up-regulated at least 2 fold in bradyzoites compared to extracellular tachyzoites (5A) or intracellular tachyzoites (5B) reveals wild-type state-specific and mutant-specific differences. Details of cluster generation are described in the materials and methods section. Y axes show expression statistics after centering and scaling. (ET), wild-type extracellular tachyzoites; (IT), wild-type intracellular tachyzoites; (B24), (B36), (B48) and (B72), wild-type parasites after 24 h, 36 h, 48 h and 72 h of bradyzoite differentiation conditions respectively; (0 h), mutant extracellular tachyzoites; (72 h), mutant parasites 72 h post-bradyzoite differentiation conditions.

We clustered genes that are up-regulated at least two fold in wild-type bradyzoites (B72) compared to freshly egressed wild-type extracellular tachyzoites (ET) (738 genes, to the right of the double red solid lines in Figure 2) and genes that are up-regulated at least two fold in B72 compared to intracellular tachyzoites (IT) (604 genes, below the double blue solid lines in Figure 2), (Figures 6A and 6B, respectively). Genes that are in common between these two gene sets are found in clusters 1A, 1B, 2A and 2B and represent those genes that are up-regulated in B72 compared to both ET and IT (i.e., the core set of genes induced by B72), while genes that are not in common between these two gene sets are found in clusters 3A and 3B and represent the bradyzoite specific genes that are differentially expressed between ET and IT (Dataset S2 contains the Gene Ontology terms for each gene in each cluster).

Clusters 1A and 1B include genes that are induced during the early phase of differentiation; are unchanged between ET and IT, are moderately induced 24 h, 36 h, and 48 h post bradyzoite induction (B24, B36, B48) and are highest in expression level at 72 h post induction (B72). These two gene clusters include many previously described bradyzoite-specific genes, including BAG1, Enolase 1, LDH2, p18, and cyst matrix protein [24], [30]–[34]. Clusters 2A and 2B include genes that are induced during the later phase of differentiation; are unchanged among ET, IT, and B24–48, but are induced at least 2-fold in B72. The genes in clusters 1A, 1B, 2A, and 2B show little to no response in the mutants at 72 h, suggesting that the disrupted loci in these mutants are likely to be involved in the early steps of bradyzoite differentiation.

The bradyzoite specific genes that are differentially expressed between ET and IT are found in clusters 3A and 3B. Cluster 3A includes genes that are induced upon host cell invasion (ET to IT) and during the early phase of differentiation (B24–48) but expression levels begin to drop 72 h post-induction (B72). In cluster 3A, mutants 12K and 13P exhibit expression levels comparable to wild-type at time 0 h but after 72 h of induction, expression levels are more comparable to wild-type intracellular tachyzoites (IT) rather than bradyzoites (B72). In contrast, the expression pattern of cluster 3A in mutants B7, 11P, 11K, 7K and P11 is affected at time 0 h and 72 h. Cluster 3B contains genes that are up-regulated in B72 compared to IT but are relatively unchanged between B72 and ET. The expression of these genes is affected at time 0 h and 72 h in all the mutants.

Interestingly, 20.m03865 (TGME49_004420), a *T. gondii* homolg of *Cryptosporidium* oocyst wall protein (COWP) that is disrupted in mutant 7K, is found in cluster 3B along with three other oocyst wall protein genes. 20.m03865 belongs to a multigene family of proteins that are predicted to have a structural role and contribute to apicomplexan survival during environmental stress [35]. Given that these genes are up-regulated in B72 and ET compared to IT suggests that these proteins may also play a protective role against stress in extracellular tachyzoites.

The SAG-related sequences (SRS) are involved in parasite persistence and host immune evasion [6]. There are 38 SRS genes that are up-regulated at least 2-fold in B72 compared to ET and IT (Figure 6, clusters 1A, 2A, 1B and 2B) where 26 out of 38 SRS genes are up-regulated early in the differentiation pathway (Figure 6, clusters 1A and 1B) while the remaining SRS genes are up-regulated 72 h post bradyzoite induction (clusters 2A and 2B). There are only 4 SRS genes that are up-regulated in B72 and IT compared to ET (SRS 20A, 20C 30A, and 47E).

### A subclass of differentiation mutants show a delayed transcriptional response to egress

**Figure 7:**
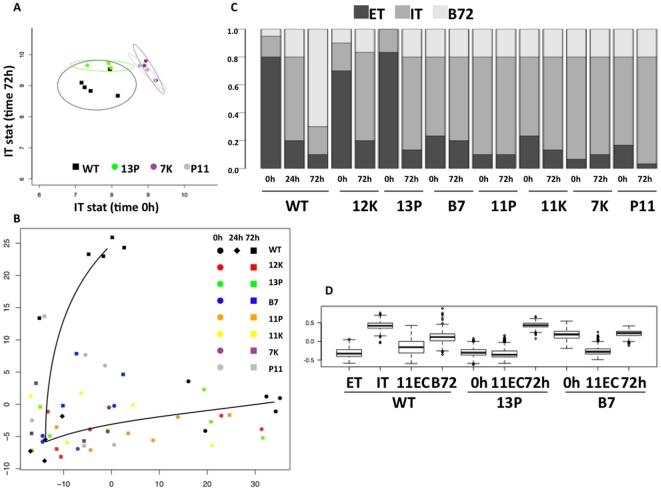
Some mutants exhibit an intracellular tachyzoite expression profile upon host cell egress. A) Analysis of intracellular tachyzoite gene expression in wild-type and mutants illustrates and quantifies differences among mutants (and between wild-type and mutants). The IT stat (see text) at time 0 h was plotted against the IT stat at time 72 h for wild-type replicate samples and replicate samples of 3 representative mutants (13P, 7K and P11). B) Principal component analysis (PCA) shows the mutant parasite sample positions in the wild-type transition from extracellular tachyzoites (black circles) to intracellular tachyzoites (black diamonds) to bradyzoites 72 h post induction (black squares). C) State modeling identified the amount to which each parasite line consisted of each of the 3 pure states (ET, IT, B72). The average proportion of each of the pure states was plotted for each sample replicate of each parasite line at each indicated time point. D) Cluster analysis of genes that are up-regulated at least 2 fold in IT compared to ET. (ET), wild-type extracellular tachyzoites; (IT), wild-type intracellular tachyzoites; (11EC) extracellular parasites 11 h after egress of host cells under tachyzoite conditions; (B72), wild-type parasites 72 h post bradyzoite induction conditions; (0 h), freshly egressed mutant extracellular tachyzoites; (72 h), mutant parasites 72 h post bradyzoite induction.

The DE plots (Figure 5) for mutants B7, 11P, 11K, 7K and P11 show a horizontal-shaped profile suggesting a global disruption in gene expression while mutants 12K and 13P show a sideways Y-shaped profile suggesting that there is a subset of genes that have similar behavior between these latter mutants and wild-type. Given that both the DE scatter plots (Figure 5) and the cluster analysis of bradyzoite up-regulated genes (Figure 6) suggest two mutant subclasses, we took a closer look at the genes that are up-regulated at least 2-fold in IT compared to ET. We designed a statistic, that we have called the ’IT stat’, which is the average expression level for all IT genes (IT - ET ≥1, 609 genes; located above the double solid green lines in Figure 2). We calculated the average expression level for the IT gene set in each parasite line at time 0 h and 72 h post bradyzoite induction, and then plotted the IT stat for each line/time point on an ellipse plot. We plotted the IT stat for replicate samples of wild-type and three mutants, representative of the two mutant subclasses (i.e., subclass 1,12K and 13P; and subclass 2, B7, 11P, 11K, 7K and P11), (Figure 7A).

The ellipse plot shows that at time 0 h, mutant 13P has an IT stat similar to wild-type parasites (∼7–8 IT stat). However, after 72h of bradyzoite induction, mutant 13P shows a ∼2 fold increase in the IT stat compared to wild-type parasites. In contrast, mutants, 7K and P11, exhibit an IT stat significantly different from wild-type parasites both under tachyzoite conditions (0 h) and bradyzoite conditions (72 h), i.e., the mean expression level of IT genes is ∼9 at both 0 h and 72 h (Figure 7A). The width of each ellipse reflects the amount of variation (analogous to the standard deviation) within each sample group, the replicate samples. Although the overall variance is larger, the IT stat for mutant 12K is similar to 13P and the IT stat for mutants 11K, B7 and 11P is similar to 7K and P11 (data not shown). We additionally performed multivariate analysis (MANOVA) to test the null hypothesis that the expression profiles for all mutants were exactly the same and the resulting p-value (<0.03) rejects the null hypothesis indicating that mutant subclasses are significantly different from each other with respect to the genes that are up-regulated in wild-type intracellular tachyzoites (IT - ET ≥1, 609 genes); consistent with the DE plots (Figure 5) and cluster analysis (Figure 6).

Principal Component Analysis (PCA) captures a high proportion of the variation across genes with a small number of parameters, permitting to identify which samples behave most like each other, and most different from each other. We performed principle component analysis and imposed a trend line for the wild-type samples only, in order to visualize the trend of the transition from tachyzoites to bradyzoites and where each mutant lies in relation to the wild-type path. Consistent with the results of gene clustering (Figure 6), PCA suggests that mutants exhibit whole genome expression comparable to intermediate points along the wild-type path from ET (black circles, lower right), through IT (black diamonds, lower left), to B72 (black squares, upper left) (Figure 7B). That is, although some variation exists within each mutant sample group, all of the samples fall along the normal differentiation pathway. At time 0 h, mutants 12K (red circles) and 13P (green circles) fall near the wild-type extracellular tachyzoites (black circles), but after 72 h of induction, these mutant lines (red (12K) and green (13P) squares) fall closest to the wild-type intracellular tachyzoites (black diamonds). In contrast, mutants B7 (blue), 11P (orange), 11K (yellow), 7K (purple), and P11 (grey) fall closest the wild-type intracellular tachyzoites (black diamonds) at times 0 h and 72 h.

A bar plot of the proportion of each pure wild-type state (see material and methods for pure state calculations) for each mutant parasite line, at time 0 h and 72 h, shows that some mutants (B7, 11P, 11K, 7K and P11) have a higher proportion of the IT state (grey bars) at time 0 h and 72 h, while other mutants (12K and 13P) exhibit wild-type-like proportions of the ET state (black bars) at time 0 h, but after 72 h of bradyzoite induction these mutants exhibit an expression profile more comparable to wild-type intracellular tachyzoites than bradyzoites (white bars) (Figure 7C).

Interestingly, mutants 12K and 13P are able to switch back and forth between the extracellular and intracellular tachyzoite states, while mutants B7, 11P, 11K, 7K and P11 appear to be ‘stuck’ in the intracellular tachyzoite state regardless of stimulus. In order to confirm this observation, we performed an experiment where we monitored the parasites as they started to egress, harvested them when at least 80% of the monolayer was lysed (time 0 h), and we also harvested parasites 11 h post egress (time 11 h). We compared one of each subclass of mutants with wild-type parasites; mutants 13P and B7 were chosen as representative mutants for the two different subclasses. The expression profile for the intracellular tachyzoite gene set (IT - ET ≥1, 609 genes) for mutant 13P (0 h and 11 h post-egress) is similar to the wild-type time points 0 h and 11 h, and no difference was observed between time 0 h and 11 h (Figure 7D). Interestingly, the expression profile for freshly egressed (0 h) mutant B7 more closely resembles wild-type intracellular tachyzoites, but at 11 h post-egress, the B7 gene expression profile returned to the wild-type freshly egressed (0 h) extracellular expression pattern (Figure 7D). Mutant B7 appears to be delayed in switching to the extracellular gene expression pattern (i.e., the ET state), in contrast to mutant 13P, that has already switched to the ET state at the moment of egress. These results suggest that the subclass 2 mutants have a delayed transcriptional response to egress.

### State modeling captures hidden variation between parasite lines to reveal genes that likely play key roles during bradyzoite differentiation

In addition to the genes identified by the insertional mutagenesis screen (Table 2), we searched for additional genes that are likely to play a mechanistic role in regulating bradyzoite differentiation. Sorting genes based on differential expression only served to describe differences in the extent to which each mutant population is ‘leaky’ (i.e., the disrupted loci caused large defects in bradyzoite formation but did not completely abolish this process). This complicates the search for genes involved in the switching mechanism because end state bradyzoite markers are both the most differentially expressed genes in the mutants in response to bradyzoite induction conditions and simultaneously the most differentially expressed between wild-type and mutant parasites. To overcome this challenge, we developed a mixture model in which each mutant parasite population is composed of differing proportions of the 3 wild-type states. This allowed for the identification of outlier genes (i.e., genes that are hidden among the large number of genes whose expression is affected) [36].

The mixture model describes each mutant using the resulting proportions of each wild-type state (Figure 7C), and genes that could not be accounted for in the model were revealed (Table 4) [36]. These genes likely play key roles in the switching mechanism. The residual values indicate how each gene in each sample differs from the wild-type states during bradyzoite differentiation (contributing in part to the distance of the mutant samples from the trend line in Figure 7B). A residual value close to 0 for a given gene in a given sample means that gene is expressed at very similar levels in the mutant compared to wild-type (contributes to the sample sitting close to the trend line in Figure 7B). Likewise, if the residual value is large (either positively or negatively) then that gene is expressed differently in that mutant compared to wild-type. The expression of some of the genes in Table 4 was also assessed by real-time PCR, which confirms these genes are expressed differently in mutant 7K (Figure S2).

**Table 4:** Top ten genes for which expression level in mutants cannot be accounted for by the three wild-type states.

Gene ID		Gene Name
ToxoDB r4.3	ToxoDB r5.3	
551.m00236	TGME49_108060	Hypothetical
44.m02759	TGME49_033000	Supt5h
113.m00800	TGME49_097840	DNA primase, large subunit
41.m02959	TGME49_022030	Hypothetical
55.m05116	TGME49_064230	Hypothetical
76.m01615	TGME49_085730	TBC domain-containing
59.m03523	TGME49_070580	HECT type ubiquitin ligase
52.m03443*		Hypothetical
38.m01105	TGME49_019240	Hypothetical
583.m05466	TGME49_112140	Hypothetical

*52.m03443 does not have a release 5 prediction which indicates that the gene model has changed.

**Figure 8:**
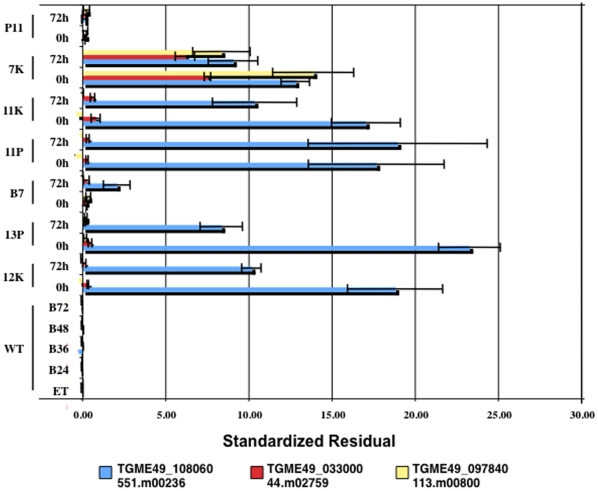
The standardized residual values for the top three genes in Table 4. Standardized residuals represent how different the observed expression of a given gene differs from the expected expression (based on wild-type state modeling). A residual value close to 0 means that the expected value is similar to the observed value. (ET), freshly egressed wild-type extracellular tachyzoites; (B24), (B36), (B48) and (B72), wild-type parasites after 24 h, 36 h, 48 h and 72 h of bradyzoite differentiation conditions respectively; (0 h), freshly egressed mutant extracellular tachyzoites; (72 h), mutant parasites 72 h post bradyzoite induction.

Standardized residuals for the top 3 genes (Figure 8) shows anomalous gene behavior between mutant and wild-type parasites, as well as between mutants. For example, the supt5h and DNA primase genes (44.m02759, TGME49_033000 and 113.m00800, TGME49_097840, respectively) are expressed at very different levels only in mutant 7K, these genes have large residual values in the 7K line (0 h and 72 h) but close to zero in the rest of the parasite lines (Figure 8, red and yellow bars, respectively). Likewise, a hypothetical gene (551.m00236, TGME49_108060) is highly expressed in all mutant lines except for mutants B7 and P11 (blue bars); an example of a candidate gene for future study.

## Discussion

Genome-wide microarray analysis suggests a novel extracellular state within the tachyzoite stage. Whole genome expression variation among extracellular tachyzoite (ET), intracellular tachyzoite (IT), and bradyzoite (B72) sample groups is equally distinct with respect to the number of differentially expressed genes and the magnitude with which the genes are differentially expressed (Figures 1 and 2), leading us to propose a novel ET “state”. Since a significant part of the cell cycle and stage conversion has been shown to be transcriptionally controlled [16], [37], it argues that the distinct transcriptional profile of ET is of biological relevance. Analysis of ontology annotation associated with differentially expressed genes is consistent with differences in organellar function (e.g., rhoptries, Table 1). Additional evidence suggests that extracellular parasites have genuinely different cell physiology and behavior, for instance, *T. gondii*'s main source of energy, the glycolytic enzymes, relocate from the cytosol to the pellicle when parasites egress from the host cells. The glycolytic enzymes remain associated to the pellicle during extended extracellular incubation and do not relocate back to the cytosol until the parasites have completed invasion of a new host cell [38]. It is possible that a fraction of the transcriptional response to egress is important for the ability of parasites to re-invade a new host cell. Taken together, these observations strongly suggest that the ET state is biologically relevant.

Recently, Behnke et al., analyzed the cell cycle transcriptome of the tachyzoite stage and showed that 2,833 genes exhibit a cell cycle expression pattern with significant peaks of mRNA abundance separated by an interval of ∼7–8 hours [37]. These 2,833 genes fall into two predominant subtranscriptomes with peak mRNA abundance in G1 phase (1146 genes) and S/M phase (1493 genes). These cell cycle regulated genes are different from the genes differentially expressed between IT, ET and B72, which suggests that the differential expression observed here is not a consequence of the redistribution of parasites among phases of the cell cycle.

On a different study, Behnke et al., reported that the expression of 267 genes were up or down-regulated by Compound 1 treatment [16]. Comparing these 267 genes with our set of differentially expressed genes by CO2 starvation treatment, shows that ∼half of these genes are differentially expressed under both conditions (Table S2). These common genes are likely to be at the core of the bradyzoite differentiation pathway since they are differentially expressed in different bradyzoite induction conditions (compound 1 and CO2 starvation) and different *T. gondii* strains. Our experiments were carried out with a type I strain while Behnke et al., used type II and III strains [16]. The rest of the genes that are not common between different treatments are likely to represent stress response genes (Table S2).

The disrupted locus in mutant P11 is predicted to encode a serine/arginine rich-4 (SR) splicing factor (Table 2). We hypothesize that this mutant is unable to correctly splice one or more transcripts involved in the regulation of bradyzoite differentiation. SR proteins regulate key processes including cell differentiation, for example, disruption of an SR protein (nSR100) prevents neural cell differentiation [39].

The insertion site in mutant B7 is just upstream from a gene predicted to be a DNA replication factor (Table 2), however, rapid amplification of cDNA ends (RACE) shows that the disrupted locus encodes a transcript with no obvious open reading frame (data not shown). The ToxoGeneChip does not include a probe for this transcript as there is no gene prediction at this locus. We hypothesize that this transcript encodes a non-coding RNA involved in the regulation of bradyzoite differentiation [40]. Frankel et al., isolated 39 mutants with a ∼10 fold reduction in the number of cysts per brain compared with infections with wild-type parasites [41]. Interestingly, one of these mutants, mutant 29C3, has an insertion very close to the insertion observed in mutant B7, i.e., the same transcript disrupted in mutant B7 is disrupted in mutant 29C3 at a different position. The fact that two different screens in two different laboratories isolated mutants with similar phenotype and the same transcript disrupted, strongly suggests that this locus is very important for cyst formation.

Solubilization evidence suggests that the members of COWP family of proteins form multimeric complexes mediated by disulfide bridges [42]. We hypothesize that the knock-out of one of these COWP members in mutant 7K, prevents correct cyst wall formation which in turn stops the differentiation process.

All seven mutants show defects in the expression of the end state bradyzoite genes.

Interestingly, the genes that fail to respond to induction conditions fall into two different subtypes: genes that are “not on” (Figure 6, clusters 1A, 2A,1B, 2B) in the mutants and genes that are “already on” (Figure 6, clusters 3A and 3B) in the mutants. Genes that are “not on” remain at unchanged basal levels in mutants at time 0 h and 72 h and correspond to genes that are induced in the bradyzoite end state. In contrast, the genes that are “already on” are expressed at abnormal high levels under tachyzoite conditions (time 0 h) and remain unchanged after 72 h of induction. It is conceivable that in order for bradyzoite formation to occur, a specific set of genes must be poised in the “off” position awaiting a signal that causes the induction of these genes, initiating the bradyzoite differentiation cascade. Much like a pinball game, the ball must be “poised” in the down position so when the handle is pulled the ball flies up and enters the game. If the ball is already in the up position then pulling the handle will not initiate the flight of the ball. Future experiments that test the “pinball” hypothesis for the “already on” gene set are warranted.

We described two mutant subclasses: 1) mutants 12K and 13P that have an expression profile similar to wild-type at time 0 h but similar to intracellular tachyzoites after 72 h of bradyzoite induction; and 2) mutants B7, 11P, 11K, 7K and P11 that exhibit an expression profile comparable to intracellular tachyzoites at both time 0 h and 72 h. While subclass 1 mutants are able switch between the intracellular tachyzoite and extracellular tachyzoite expression profiles at the same rate as wild-type, subclass 2 mutants have a defect in the transition from intracellular tachyzoites to extracellular tachyzoites: they show a delayed transcriptional response to egress. The fact that these mutants were selected for failure to differentiate into bradyzoites and also show a defect switching to the extracellular state suggests a common link between these two cellular processes. For example, one similarity between the intracellular tachyzoite to bradyzoite and the intracellular tachyzoite to extracellular tachyzoite transitions is that both processes require that the parasites slow down their growth rate. While intracellular tachyzoites are committed to cell growth and replication, bradyzoite invasion is essential for transmission through the oral route and extracellular tachyzoite invasion is essential for parasite dissemination throughout the host.

A mixture model identified the putative supt5h protein (44.m02759, TGME49_033000) and the DNA primase (113.m00800, TGME49_097840) genes as having highly anomalous expression in the mutants and, thus, may be involved in the switching mechanism. The supt5h gene is predicted to be a transcription elongation factor, therefore, it is possible that expression of this gene is associated with control of developmental progression in *T. gondii*. The DNA primase gene is predicted to be involved in the synthesis of RNA primers during lagging strand DNA replication. Considering that *T. gondii* has to slow down its cell growth rate in order to form bradyzoites, it is not surprising that genes involved in DNA replication are implicated in the developmental transition. These genes are two candidates to further explore the mechanistic switch during bradyzoite formation.

In conclusion, we identified a novel state in the normal asexual cycle of *T. gondii*, and propose that the asexual cycle consists of 3 distinct states; extracellular tachyzoites, intracellular tachyzoites and bradyzoites. We describe the identification of 7 insertional mutants that are unable to undergo normal bradyzoite differentiation. Our data suggests a link between the transition from intracellular tachyzoites to either extracellular tachyzoites or bradyzoites. In addition to the genes identified by the insertional mutagenesis screen, a mixture model allowed us to identify a small number of genes for which their expression pattern could not be accounted using the 3 wild-type states, genes that may play a mechanistic role in the differentiation cascade. This study also illustrates the power of combining a genomic approach with a genetic approach, opening up the foundation for several future studies, such as analyzing the role of the genes revealed by the state modeling or exploring a possible link between the transition to bradyzoites and the transition to the extracellular state. In addition, this work could potentially lead to the identification of novel targets for drug development.

## Materials and Methods

### Parasite growth and differentiation

RH strain *T. gondii* tachyzoites (wild-type and mutants) were maintained by serial passage in primary cultures of Human foreskin fibroblast (HFF) cells, as described previously [43]. Parasites were induced to differentiate into bradyzoites in low [CO_2_], resulting in pyrimidine starvation [17]. CO_2_ depletion was accomplished by inoculating tachyzoites in host cells in minimal essential medium without NaHCO_3_ but containing 25 mM HEPES. The low CO_2_ bradyzoite induction method was used for all bradyzoite differentiation experiments described throughout this report.

### Selection of insertional mutants

The selection screen has been described in [17]. Briefly, insertional mutagenesis was carried out by transfection using linearized plasmid pDHFR*-TSc3 or pDHFR*-TSc3ABP [44], [45]. These vectors integrate throughout the parasite genome by non-homologous recombination, conferring resistance to 1 mM pyrimethamine.

### Identification of disrupted loci in the mutants

#### Inverse PCR

Southern blot analysis with different restriction enzymes that cut within the mutagenesis vector (pDHFR*-TSc3 [44]) revealed bands that extend from the vector sequence into the parasite genome and were short enough to PCR. The inverse PCR technique is described in [36]. Briefly, restriction enzymes were used to cut the genomic DNA and fragments were ligated in a dilute solution in order to circularize the DNA. Primers oriented in opposite directions within the mutagenesis vector were used to amplify the genomic region flanking the insertion site. The inverse PCR products of the expected size were then sequenced and aligned using BLAST in ToxoDB. To confirm the results of the inverse PCR, southern-blots were carried out.

#### Plasmid rescue

An alternative method to inverse PCR, is plasmid rescue [44]. This technique is carried out in the same way as inverse PCR except that instead of a PCR amplification, the circularized DNA fragments are transformed into *Escherichia coli* bacteria and selected with the selectable marker present in the mutagenesis vector.

### Immunofluorescence assays

HFF cells were grown to confluence on glass coverslips in 6 well plates. The confluent HFF cells were infected with equal number of mutant and the parental wild-type (WT) parasites. After 72 hours of bradyzoite induction using CO_2_ starvation, the coverslips were fixed with 3.7% formaldehyde in phosphate-buffered saline (PBS) for 10 minutes at room temperature, permeabilized with 0.25% Triton-X-100 in PBS for 15 min at room temperature and blocked with 1X PBS 1% bovine serum albumin (BSA) for 30 min at room temperature. The coverslips were incubated for 1 h with antibody against BAG1, followed with 488 (goat anti-rabbit immunoglobulin G conjugated to Alexa fluor, Molecular Probes), and tetramethyl rhodamine isothiocyanate (TRITC)-labeled Dolichos biflorus lectin (DL) (Sigma-Aldrich). The slides were analyzed using a Leica DMIRE2 fluorescence microscope (Leica Microsystems) and images were captured using Improvision Openlab 4.0.2 software (Improvision). The samples were examined at 100X magnification and 100 vacuoles were counted. The number of vacuoles positive for BAG1 and DL staining were expressed as % positive vacuoles from duplicate samples. In addition, the number of parasites/vacuole was recorded simultaneously and the average number of parasites/vacuole was determined.

### Quantitative real time RT-PCR

Total RNA was extracted using RNeasy kit (Qiagen). DNase treated RNA (2 µg) was subjected to the RT reaction. The primer and probe sets (Sigma Genosys) were designed using Primer Express software, version 3.0 (Applied Biosystems). Real-time PCR was performed using a 7500 Fast Real-Time PCR system (Applied Biosystems). The constitutively expressed *T. gondii* DHFR gene was used as the endogenous control. Primers and probes are listed in Table 5.

**Table 5:** Primers and Probes for quantitative real-time PCR.

Gene	Primer (5′-3′)	Probe (5′-3′)
**DHFR**	Forward: CGCGAGCAAAAGGAACTGAReverse: CAATGAGATCAAGGTACTGGAATTCTT	6CCGTTCCGCATGTTCACTTTAGAGGC[BHQ1]
**BAG1**	Forward: TCCCATCGACGATATGTTGTTCReverse: CCACGTGATGTCCTCCATCA	6AGACGGCCCTCACCGCAAACG[BHQ1]
**ROP- 23**	Forward: CCAAGCGAGGCGTATTCGReverse: AAGTAGCGGCTGGAGTTGGA	6AGACCGCTTAATGCTTCCTCTCGATCTGC[BHQ1]
**113.m00800**	Forward: TCTTCCGCATCTTTCAACGAReverse: GTTGCACCGCGACCTCTT	6TCCCGTGAAGGCCT[BHQ1]
**41.m02959**	Forward: GAGCTCCGTCTTGCATTGCReverse: CTCGATGCCGAGACATGCTA	6CTTTACCAGCAACCGTG[BHQ1]
**38.m01105**	Forward: GCCCCGACCCCATTGTReverse: CACACCACACGAGCACTGTTC	6CAGTATGTCTTGCTTCGGA[BHQ1]

### Microarray hybridizations

Total RNA was isolated from parental and mutant extracellular tachyzoites (ET) (freshly egressed), intracellular tachyzoites 24 hours after invasion (IT), or bradyzoites (B72) (grown under differentiation conditions for 72 hours) using the RNeasy mini kit (Qiagen). A minimum of three independent experimental inductions was carried out for each parasite line.

### Microarray Analysis and Statistics

All data are MIAME compliant and are archived at NCBI GEO under Series GSE23174.

### Overview (Figure 9)

**Figure 9:**
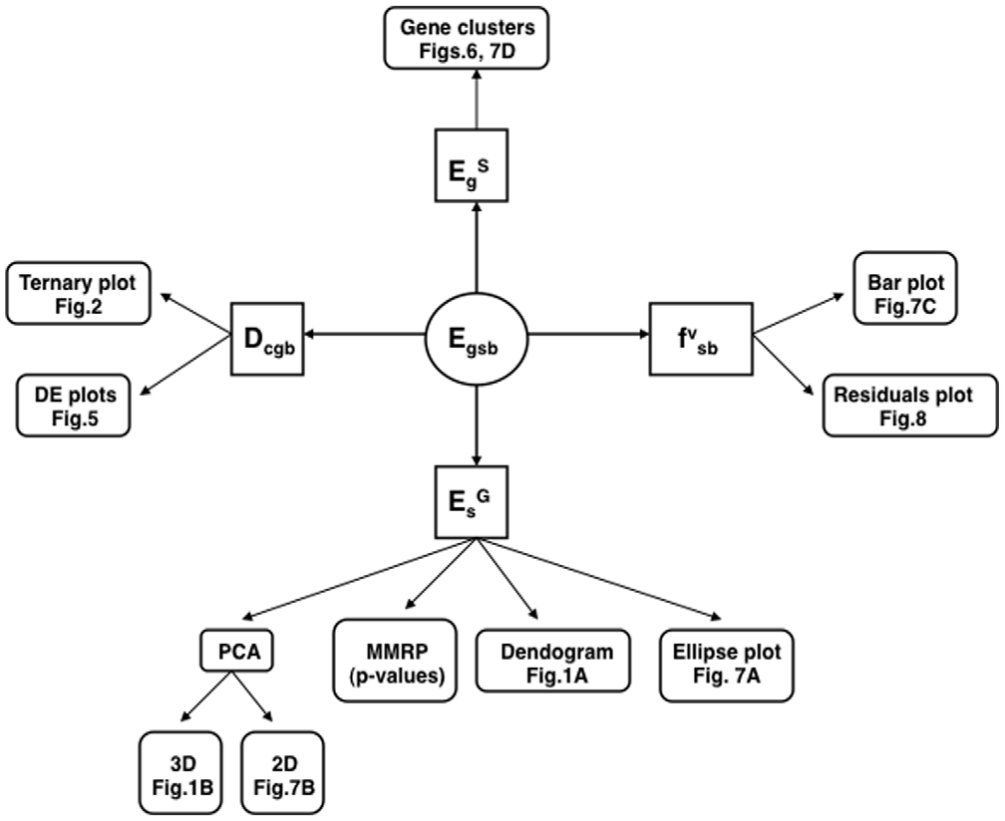
Data types involved in microarray analysis. The gene by sample expression matrix (E_gsb_) is used to do 1) sample based analysis (E_s_^G^) where the sample vector for each gene is used to generate PCA, dendogram and ellipse plots (and associated p-values), 2) gene based analysis (E_g_^S^) where the gene vector for each sample is used to generate gene clusters, 3) differential expression for contrasts (D_cgb_) to generate ternary and DE plots, and 4) mixture modeling (f^v^_sb_) to generate state model box plots and residuals.

Raw GeneChip data (one DAT file for each chip or, equivalently, sample) includes a collection of images, one for each probe and chip. Each image is summarized by Affymetrix GCOS software using one probe intensity (in CEL files, one per chip). We calculated a summary measure of expression, 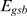, for each probe set (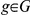, where *G* is the set of probe sets or, informally, genes), sample (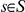, where 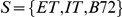 is the set of samples in a batch) and batch (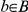, where *B* is the set of batches or, equivalently, experiment dates) using the Robust Multichip Average (RMA) method of Speed and coworkers [46], [47]. Calculations were performed using R [48] and BioConductor [49] tools, in particular, the aroma.affymetrix package of Bengtsson [50].

Analysis based on 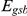 included 1) exploratory multivariate analysis of sample profiles,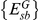 (each sample is represented by a vector describing the expression statistics for all probe sets), 2) exploratory multivariate analysis of gene profiles, 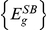 (each probe set is represented as a vector describing the expression in all samples in all batches), and 3) differences based on contrasts,



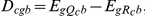
where 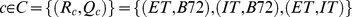 and 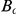 is the set of batches compatible with comparison 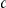. We averaged 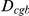 over batches



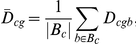
and over a set of genes, 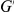,
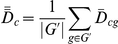



### Analysis of 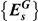


Sample-sample distances were calculated using the Euclidean distance and expressed as the root mean square deviation (RMSD). The RMSD expresses the differences between two samples in a way that can be evaluated in the context of intuition about the change in a single gene, for example, a difference of 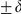 in the expression of every gene would yield an RMSD of 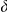. Hierarchical sample clustering was performed using complete linkage (the hclust procedure in the R stats package, with default arguments).

Principal component analysis was used to reduce the 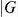-dimensional vectors describing samples to two or three uncorrelated random variables that capture a substantial proportion of the variation between samples. Principal component analysis was performed using the princomp procedure in the R stats package.

### Analysis of 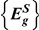


The dissimilarity between two probe sets (labeled 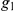 and 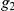) with respect to their expression profiles (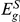 and 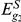) is 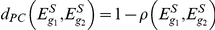
where 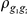 is Pearson's correlation coefficient. Probe sets were assigned to classes using the k-means algorithm [51], implemented in the k-means procedure in the R stats package.

### Analysis of 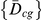


Given three sample conditions (*B72*, *ET*, and *IT*), the ternary plot provides a two-dimensional representation of the three contrasts, 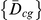. Each of the comparisons is assigned a color (red, green, and blue, respectively).

Each probe set is represented by a point in the plane 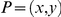, where



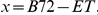


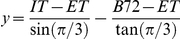



Then



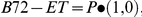





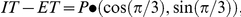





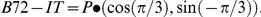



With this representation the differences 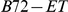, 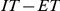, 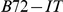 associated with a gene can be read off the ternary plot as projections of 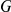 along the dashed lines at angles 0, 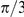, and 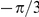 with respect to the horizontal axis, respectively.

Pairs of solid lines bound sets of probe sets for which the differential expression is less than or equal to 2-fold (up or down) with respect to the comparison indicated by the color. The six solid lines define 19 elementary regions in the plane, each of which is labeled with the number of probe sets it contains. For example, 375 probe sets exhibited IT expression within 2-fold of ET expression while B72 expression is 2-fold greater than either IT or ET.

Two independent contrasts, 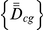, are represented for a set of mutants (Figure 7A) along with an ellipse representing the sample group covariance.

### Mixture Model

The wild type samples were fit to a three-component mixture model,
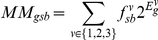



Where 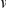 indicates a mixture component, 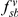 is its proportion in the sample indicated by 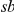, and 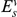 is the expression of gene 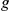 of the pure 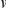 state. More precisely, we minimized the weighted sum of squared residuals 
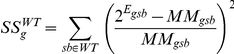
with respect to 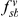 and 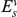. The 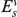 were then used to find mixture proportions for the mutants by minimizing



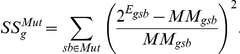
with respect to 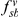. Genes were ordered based on 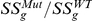. For the genes reported in Table 4 and Figure 8 the root mean square deviation of the mutant samples is more than 10 times that of the wild-type samples, suggesting a negligible false discovery rate (residuals for the top three genes in Figure 8).

### Gene Ontology Analysis

We used Fisher's exact test to test for significance of contingency tables of GO term and differential expression gene sets.

## Supporting Information

Figure S1 Quantitative real time PCR (Taqman) results correlate positively with the microarray results. Total RNA was extracted from wild-type and mutant parasites, subjected to reverse transcription to obtain cDNA, and real time PCR carried out using primers from BAG1 and ROP23 genes (see Experimental procedures). The values indicate the relative gene expression (RE) levels normalized to the expression levels of DHFR (endogenous control). The error bars represent the standard deviation (SD) from duplicate experiments. (WT.T) wild-type tachyzoites; (WT.B) wild-type bradyzoites; (12K.T), tachyzoites of mutant 12K; (12K.B), bradyzoites of mutant 12K.
(1.56 MB TIF)

Figure S2 Quantitative real time PCR (Taqman) results correlate with the microarray results. Quantitative real time PCR was carried out as described above (Fig. S1) using primers for 3 genes described in Table 4 (113.m00800, 41.m02959 and 38.m01105). These results are compared with mutant 7K, because these genes show anomalous behavior in this mutant (see Fig. 8). (WT.T) wild-type tachyzoites; (WT.B) wild-type bradyzoites; (7K.T), tachyzoites of mutant 7K; (7K.B), bradyzoites of mutant 7K.
(1.56 MB TIF)

Figure S3 PCA histogram shows the contribution of the first 10 principle components for the wild-type sample replicates.
(1.56 MB TIF)

Figure S4 Southern-blots show that each mutant has been disrupted in a different locus. Genomic DNA was isolated from wild-type and the seven mutant parasite lines, digested with BamHI and subjected to Southern analysis using a 32P- labeled fragment derived from the insertional mutagenesis vector pDHFR*-TSc3.
(1.56 MB TIF)

Figure S5 IFAs carried out at 12 h post bradyzoite induction. Parasites were stained with antibodies against the cell cycle markers IMC1 (green), Centrin (red) and dapi (blue).
(1.56 MB TIF)

Dataset S1 GO terms for wild-type state-specific gene sets; ET, IT and B72.
(0.14 MB XLS)

Dataset S2 GO terms for the genes present in each of the six bradyzoite up-regulated gene clusters.
(0.22 MB XLS)

Table S1 Expression profile of genes surrounding the insertion point in each of the mutants. Mutant 7K is shown in Table 3. Mutant P11 is not shown because it was complemented with a DNA fragment that only contains TGME49_013640 (31.m00914) and therefore, there is no doubt this gene is responsible for the phenotype observed in this mutant.
(0.04 MB DOC)

Table S2 Comparison with the results reported by Behnke et al., [16]. Expression of 267 genes up or down-regulated by Compound 1. The genes up-regulated in both our data and Behnke et al., are shown in red. The genes down-regulated are shown in green.
(0.48 MB XLS)
