# Supplementary figures and images for: Genomic Data Reveal Toxoplasma gondii Differentiation Mutants Are Also Impaired with Respect to Switching into a Novel Extracellular Tachyzoite State
Source: PLoS One. 2010 Dec 30;5(12):e14463. doi: 10.1371/journal.pone.0014463 (PMC3012682; doi:10.1371/journal.pone.0014463)

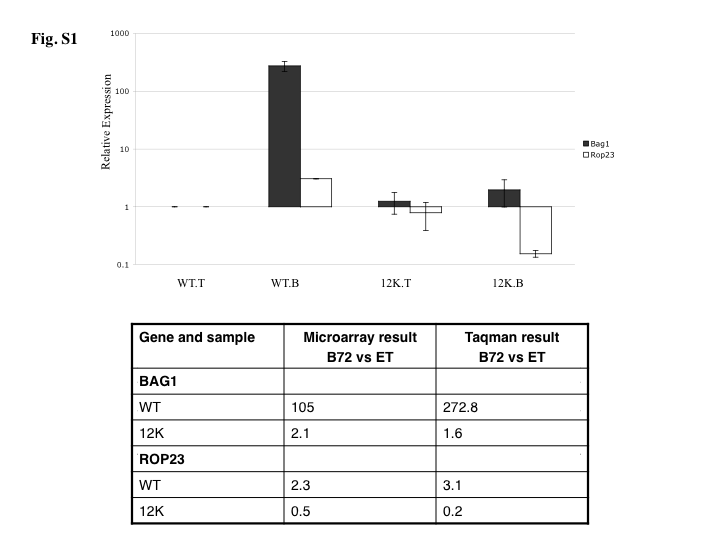

Supplement: Figure S1 — Quantitative real time PCR (Taqman) results correlate positively with the microarray results. Total RNA was extracted from wild-type and mutant parasites, subjected to reverse transcription to obtain cDNA, and real time PCR carried out using primers from BAG1 and ROP23 genes (see Experimental procedures). The values indicate the relative gene expression (RE) levels normalized to the expression levels of DHFR (endogenous control). The error bars represent the standard deviation (SD) from duplicate experiments. (WT.T) wild-type tachyzoites; (WT.B) wild-type bradyzoites; (12K.T), tachyzoites of mutant 12K; (12K.B), bradyzoites of mutant 12K. (1.56 MB TIF) [file pone.0014463.s001.tif]

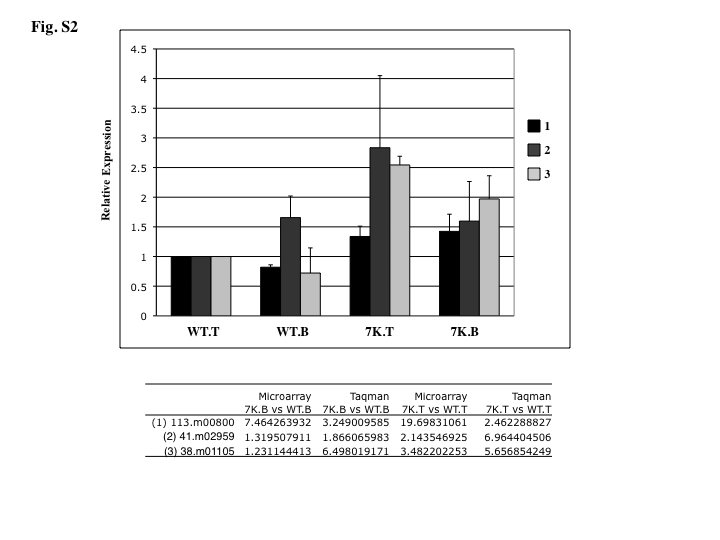

Supplement: Figure S2 — Quantitative real time PCR (Taqman) results correlate with the microarray results. Quantitative real time PCR was carried out as described above (Fig. S1) using primers for 3 genes described in Table 4 (113.m00800, 41.m02959 and 38.m01105). These results are compared with mutant 7K, because these genes show anomalous behavior in this mutant (see Fig. 8). (WT.T) wild-type tachyzoites; (WT.B) wild-type bradyzoites; (7K.T), tachyzoites of mutant 7K; (7K.B), bradyzoites of mutant 7K. (1.56 MB TIF) [file pone.0014463.s002.tif]

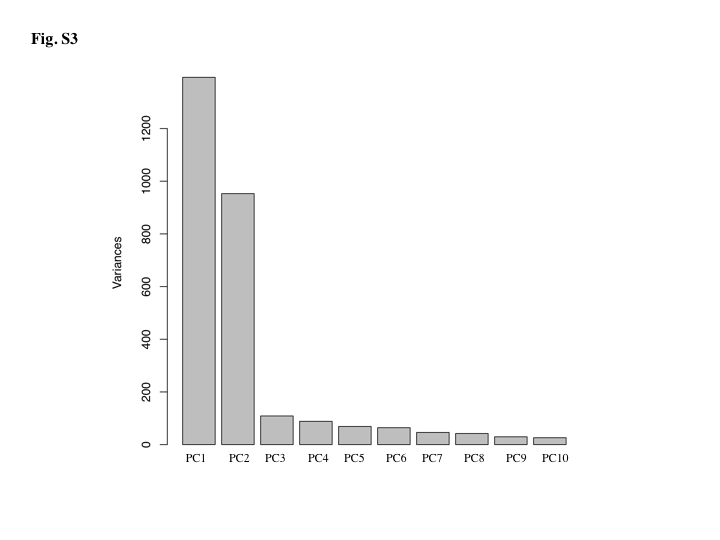

Supplement: Figure S3 — PCA histogram shows the contribution of the first 10 principle components for the wild-type sample replicates. (1.56 MB TIF) [file pone.0014463.s003.tif]

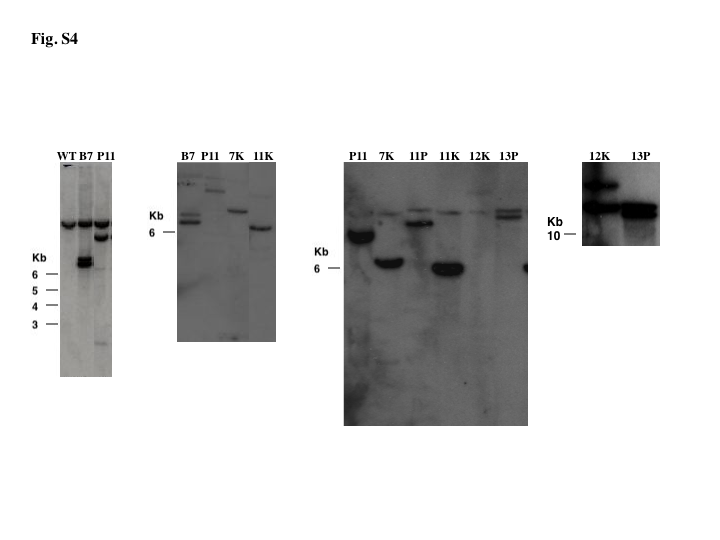

Supplement: Figure S4 — Southern-blots show that each mutant has been disrupted in a different locus. Genomic DNA was isolated from wild-type and the seven mutant parasite lines, digested with BamHI and subjected to Southern analysis using a 32P- labeled fragment derived from the insertional mutagenesis vector pDHFR*-TSc3. (1.56 MB TIF) [file pone.0014463.s004.tif]

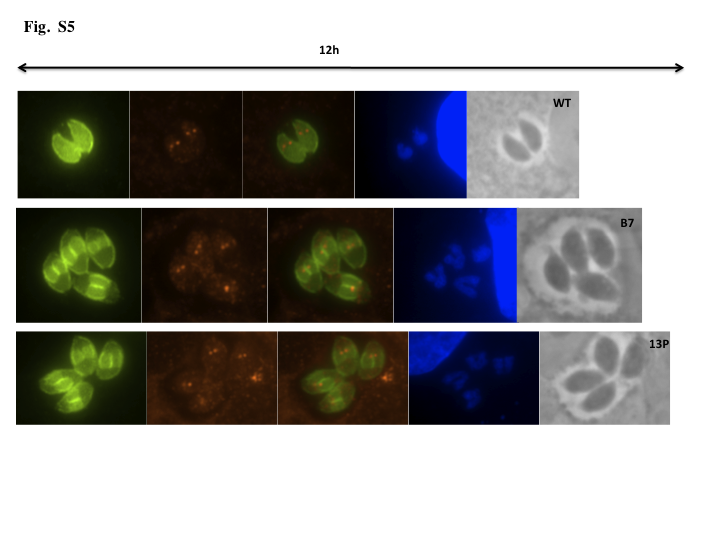

Supplement: Figure S5 — IFAs carried out at 12 h post bradyzoite induction. Parasites were stained with antibodies against the cell cycle markers IMC1 (green), Centrin (red) and dapi (blue). (1.56 MB TIF) [file pone.0014463.s005.tif]
