# Supplementary material for: Genomic Data Reveal Toxoplasma gondii Differentiation Mutants Are Also Impaired with Respect to Switching into a Novel Extracellular Tachyzoite State
Source: PLoS One. 2010 Dec 30;5(12):e14463. doi: 10.1371/journal.pone.0014463 (PMC3012682; doi:10.1371/journal.pone.0014463)
Supplement: Table S1 — Expression profile of genes surrounding the insertion point in each of the mutants. Mutant 7K is shown in Table 3. Mutant P11 is not shown because it was complemented with a DNA fragment that only contains TGME49_013640 (31.m00914) and therefore, there is no doubt this gene is responsible for the phenotype observed in this mutant. (0.04 MB DOC) [file pone.0014463.s008.doc]

**Table S1.** Expression profile of genes surrounding the insertion point in each of the mutants. Mutant 7K is shown in Table 3. Mutant P11 is not shown because it was complemented with a DNA fragment that only contains TGME49_013640 (31.m00914) and therefore, there is no doubt this gene is responsible for the phenotype observed in mutant P11. We show the difference in expression of each pair of expression statistics.

| Gene Symbol | Chr | B72-ET (WT) | B72-ET (B7) | B72-ET (11P) | B72-ET (11K) |
| --- | --- | --- | --- | --- | --- |
|  |  |  |  |  |  |
| Mutant 11P |  |  |  |  |  |
| 50.m03282 | XII | 0.180698195 |  | 0.480275575 |  |
| 50.m03283 | XII | 0.211190354 |  | -0.030777734 |  |
| 50.m07123 | XII | -0.29533661 |  | 0.091239519 |  |
| 50.m00016 | XII | -0.083816406 |  | 0.020280806 |  |
|  |  |  |  |  |  |
| Mutant B7 |  |  |  |  |  |
| 49.m00018 | VI | 0.444659976 | -0.114374285 |  |  |
| 49.m07209 | VI | -0.66687209 | -0.06338353 |  |  |
| 49.m07212 | VI | -0.587327369 | -0.260665425 |  |  |
|  |  |  |  |  |  |
| Mutant 11K |  |  |  |  |  |
| 42.m03399 | X | 0.633606962 |  |  | 0.782942357 |
| 42.m03397 | X | -0.995942678 |  |  | -0.342819469 |
| 42.m03398 | X | -0.799611507 |  |  | -0.297471922 |
